# Housefly gut microbiomes as a reservoir and facilitator for the spread of antibiotic resistance

**DOI:** 10.1093/ismejo/wrae128

**Published:** 2024-07-20

**Authors:** Dehao Gan, Zhenyan Lin, Lingshuang Zeng, Hui Deng, Timothy R Walsh, Shungui Zhou, Qiu E Yang

**Affiliations:** Fujian Provincial Key Laboratory of Soil Environmental Health and Regulation, College of Resources and Environment, Fujian Agriculture and Forestry University, Fuzhou 350002, China; Fujian Key Laboratory of Traditional Chinese Veterinary Medicine and Animal Health, College of Animal Sciences, Fujian Agriculture and Forestry University, Fuzhou 350002, China; Fujian Provincial Key Laboratory of Soil Environmental Health and Regulation, College of Resources and Environment, Fujian Agriculture and Forestry University, Fuzhou 350002, China; Fujian Provincial Key Laboratory of Soil Environmental Health and Regulation, College of Resources and Environment, Fujian Agriculture and Forestry University, Fuzhou 350002, China; Fujian Key Laboratory of Traditional Chinese Veterinary Medicine and Animal Health, College of Animal Sciences, Fujian Agriculture and Forestry University, Fuzhou 350002, China; Ineos Oxford Institute for Antimicrobial Research, Department of Biology, University of Oxford, Oxford OX1 3RE, United Kingdom; Fujian Provincial Key Laboratory of Soil Environmental Health and Regulation, College of Resources and Environment, Fujian Agriculture and Forestry University, Fuzhou 350002, China; Fujian Provincial Key Laboratory of Soil Environmental Health and Regulation, College of Resources and Environment, Fujian Agriculture and Forestry University, Fuzhou 350002, China

**Keywords:** gut microbiota, housefly, antimicrobial resistance, plasmid-borne ARGs, horizontal gene transfer

## Abstract

Arthropods, such as houseflies, play a significant role in the dissemination of antimicrobial resistance (AMR); however, their impact has often been overlooked in comparison to other AMR vectors. Understanding the contribution of arthropods to the spread of AMR is critical for implementing robust policies to mitigate the spread of AMR across One Health sectors, affecting animals and environmental habitats as well as humans. In this study, we investigated the *in situ* transfer of a *gfp*-labelled AMR plasmid (IncA/C carrying an *mcr-8* gene, pA/C_MCR-8) in the gut microbiota of houseflies (*Musca domestica*) by applying single-cell sorting, 16S rRNA gene amplicon sequencing and whole-genome sequencing. Our findings demonstrate that the pA/C_MCR-8–positive *Escherichia coli* donor strain is capable of colonizing the gut microbiome of houseflies and persists in the housefly intestine for 5 days; however, no transfer was detectable above the detection threshold of 10^−5^ per cell. The conjugative plasmid pA/C_MCR-8 demonstrated a high transfer frequency ranging from 4.1 × 10^−3^ to 5.0 × 10^−3^ per cell *in vitro* and exhibited transfer across various bacterial phyla, primarily encompassing *Pseudomonadota* and *Bacillota*. Phylogenic analysis has revealed that *Providencia stuartii,* a human opportunistic pathogen, is a notable recipient of pA/C_MCR-8. The conjugation assays further revealed that newly formed *P. stuartii* transconjugants readily transfer pA/C_MCR-8 to other clinically relevant pathogens (e.g. *Klebsiella pneumoniae*). Our findings indicate the potential transfer of AMR plasmids from houseflies to human opportunistic pathogens and further support the adoption of a One Health approach in developing infection control policies that address AMR across clinical settings.

## Introduction

Antimicrobial resistance (AMR), which has become one of the world’s biggest health problems, has the potential to render many of the current mainstay and last-resort antibiotics ineffective, posing significant challenges to the effective treatment of infectious diseases [1, 2]. AMR is a One Health problem, recognized to affect not only human health but also the health of animals and our shared environment, as many antibiotic resistance genes (ARGs) of concern in human pathogens are also prevalent in bacteria from animals and environmental habitats [3, 4]. Although the overuse and misuse of antibiotics primarily drives the emergence and dissemination of ARGs, the spread of AMR is further exaggerated by horizontal gene transfer (HGT) mediated by conjugative plasmids [5] and other genetic mechanisms, including transduction and transformation [6]. One such mechanism of HGT occurs through the gut microbiome of insects, particularly houseflies, which have been recognized as potential carriers and reservoirs for antibiotic-resistant bacteria and ARGs [7]. Understanding the role of the housefly gut microbiome in the transmission and dissemination of antibiotic resistance is critical for mitigating the spread of AMR.

Houseflies can act as mechanical vectors for the spread of ARGs, due to their ubiquitous nature and close association with human environments such as hospitals, farms, and waste disposal sites [8–10]. Recent research provides compelling evidence of the transmission of ARGs by arthropods in hospitals, indicating that ~35.4% and 15.2% of insects (ants, cockroaches, flies, moths, and spiders) obtained from a hospital in Pakistan carried the *bla*_CTX-M-15_ and *bla*_NDM_ gene, respectively, conferring resistance to the last-line drugs reserved for life-threatening infections, such as carbapenems [7]. However, current observational studies have solely focused on identifying the presence of known ARGs in those insects by use of polymerase chain reaction (PCR) or metagenomic analyses [11], limiting the ability to discern the genetic factors (i.e. plasmids) for facilitating the mobilization of ARGs to other microbiome members, including human pathogens. Therefore, it is important to determine the role of plasmids in the spread of ARGs and identify the bacterial hosts that can acquire and retain AMR plasmids in the gut microbiome of flies.

One critical challenge is how to examine the *in situ* transfer of AMR plasmids within gut microbiome and identify the plasmid hosts that can acquire and maintain AMR plasmids. The transfer of plasmid-borne ARGs (*bla*_CTX-M_ or *bla*_CMY-2_) [12] and virulence factor *stx1* [13] within the intestines of houseflies has been studied using culture-based methods. For example, Akhtar and colleagues have found that plasmid pCF10, which carryies a tetracycline resistance gene, *tetM*, was able to transfer this gene to a specific recipient strain, *Enterococcus faecalis*, within the housefly digestive tract, with transfer rates ranging from 8.6 × 10^−5^ to 5.5 × 10^−1^ per donor cell [14]. This finding suggests that houseflies may provide a favorable environment for the emergence of multidrug-resistant pathogenic bacteria through acquisition of plasmid-borne ARGs. This culture-based method typically involves using a pair of specific donor and recipient strains with known antibiotic resistance markers and the isolation of transconjugants using selective agar plating. However, because many bacteria in natural communities are not easily culturable using traditional methods [15], these culture-based experimental results may not fully represent the complexity of microbial communities present in natural environments like the intestine of houseflies and may lead to an incomplete understanding of microbial biology and genetic exchanges. By complementing traditional culture-based methods, a culture-independent method of examining HGT in complex microbiomes is to use fluorescence reporters built into IncPs (incompatibility group plasmids)which allows simplification of the isolation of target cells in a rapid, sensitive, and high-throughput manner using flow-activated cell sorting (FACS) [16–18]. The transfer potentials of several IncP-type plasmids in natural microbial communities have been examined by using this culture-independent method [19–21], which has provided better understanding of the true scale of plasmid-mediated AMR transfer in natural habitats, such as soil and wastewater microbiomes [22, 23]. However, this culture-independent method has not been widely explored in other clinically relevant plasmids or in the gut microbiome of insects, leaving a knowledge gap in understanding and tracking the plasmid transfer in different environmental settings.

In this study, we aimed to investigate the mechanisms underlying the colonization ability of the *mcr-8*–positive donor strain in the housefly gut, as well as the transfer dynamics and characteristics of the newly acquired plasmid hosts. By integrating the fluorescence-based methods with culture-based approaches, we determined that a focus AMR plasmid (pA/C_MCR-8) is capable of rapid transfer across the housefly gut microbiome. Furthermore, we observed a close association between plasmid pA/C_MCR-8 and *Providencia* bacteria, which share substantial genetic identity with pathogens known to cause infections. These findings provide crucial insights into the potential role of houseflies as vectors for the spread of ARGs in both clinical and environmental settings. Understanding these mechanisms is essential for developing effective strategies to combat AMR and safeguard public health.

## Materials and Methods

### Bacterial strain and growth conditions

The bacterial strains used in this study are listed in Supplementary Table S1. Bacterial strains were grown in Luria-Bertani (LB) broth (HuanKai Microbial, China) at 37°C with shaking (150 rpm) or on LB agar plates. Antibiotics were added at the following concentrations for conjugation experiments: 30 mg/l kanamycin (for donor strain MG1655 tagged with a kanamycin resistance gene *aphA*) [17, 19] and 2 mg/l colistin (for selecting transconjugants with plasmid pA/C_MCR-8 conferring colistin resistance). When measuring bacterial growth curves, bacterial optical density (OD_600nm_) was determined by SpectraMax iD3 (Molecular Devices, United States). The bacterial growth parameters were analyzed using the Growthcurver R package (version v0.3.0), including bacterial carrying capacity (*k*), the growth rate (*r*), OD_600max_, and the area under the growth curve (AUC) [24].

### Rearing of houseflies

Housefly (*Musca domestica*) pupae were purchased from the Rongfei Eco-Tech Co (Anhui, China). Pupae were incubated at room temperature in contained cages covered with polyester netting. Emerged flies were provided with brown sugar dissolved in sterile LB broth. Both water and sugar sources were replaced daily.

### Extraction of gut microbiota from houseflies

To sterilize the outer surface and remove external contamination, houseflies were treated under UV light for 1 hour before three washings with 75% alcohol and then sterile water. The stomachs of houseflies were carefully dissected with forceps and added to sterile Eppendorf tubes containing 250 μl phosphate buffered saline (PBS) buffer (pH 7.4 PBS containing 0.1% (w/v) L-cysteine). The gut microbiomes from three flies were pooled, thereby constituting a single specimen. The gut tissues with grinder beads were homogenized using a homogenizer (KZ-II, Servicebio, China) at 30 Hz at 4°C for 2 minutes. A total of 750 μl PBS buffer was added to homogenized sample and centrifuged at 500 rpm at 4°C for 6 minutes, then the supernatant containing the extracted microbial DNA was collected into new tubes. Bacterial pellets were re-centrifuged at 6000 rpm at 4°C for 5 minutes to remove impurities and contamination from samples. Bacterial pellets were re-suspended in 1 ml PBS buffer and stored at 4°C until use.

### *In vitro* conjugation experiments

To investigate whether the plasmid pA/C_MCR-8 could be transferred within the gut microbiota of houseflies, we performed *in vitro* conjugation experiments. In brief, the overnight culture of the donor strain carrying a pA/C_MCR-8 plasmid was 1:100 diluted in fresh LB broth and incubated for 3–4 hours to reach the bacterial exponential phase and donor strain density of ~10^7^ CFU/ml. The above extracted gut microbiota was used as recipient bacteria and mixed with the donor strain at a 1:1 ratio and co-incubated at 37°C. After 16 hours of mating, the mixtures were diluted and analyzed on an Attune NxT flow cytometer (Thermo Fisher Scientific, United States) based on bacterial cell sizes and fluorescent signal threshold settings. In addition, the conjugation events were also visualized by confocal laser scanning microscopy (Zeiss Axio Imager M2, Zeiss) and analyzed by Zeiss Zen software (ZEN 2.6 blue edition).

To sort the *gfp*-expressing transconjugants by FACS, a cytoFLEX SRT cell sorter (Beckman, United States)the *gfp*-expressing transconjugant cells were sorted based on green fluorescent signals using. Prior to sorting, the mating culture was diluted in PBS buffer to ~8000 events/s and the sorting was performed using a 488 nm (50 mW) laser and a 561 nm (30 mW) laser. The detailed gating strategy is illustrated in Supplementary Figs. S1–S2. The negative control (nonfluorescent bacteria and PBS buffer) and fluorescent-positive controls (*gfp*- and *mCherry*-positive strains) were used to set the baseline fluorescent levels (Supplementary Fig. S2) and confirm that the reported numbers of positive droplets in the green fluorescent protein (GFP) gates reflect true signals attributable to GFP expression. The CytExpert SRT software v.1.1.0.10007 was used for both operating and analyzing results. In order to avoid contamination in the sorted transconjugal pools, the first sorted transconjugal cells were subjected to the second sorting. A total of 20 000 *gfp*-positive cells were collected for each replicate, followed by bacterial lysis and gDNA extraction for 16S rRNA gene amplicon sequencing. To verify the purity of sorted transconjugants, the sorted culture was plated on an LB agar plate and incubated at 37°C for 24 hours. The green colonies were visualized with TGreen Plus OSE-470L (TIANGEN, China). A total of 11 single colonies were randomly selected from agar plates and subjected to 16S rRNA gene amplicon PCR for bacterial species identification.

### *In vivo* colonization experiments

The overnight culture of the donor strain was prepared by incubation at 37°C with shaking (180 rpm) in LB broth, followed by centrifugation (5000 rpm, 10 minutes) and resuspended in 1 ml of LB broth. The bacterial suspensions were added to sugar solution as a food supply for houseflies. After the start of bacterial feeding, 10 houseflies were collected per group every day. Each fly was washed with 75% ethanol and twice with PBS buffer. The gut microbiota of each fly was collected, as described above. The presence of *mCherry*-positive straining was determined with an Attune NxT flow cytometer (Thermo Fisher Scientific, United States).

### Taxonomy analysis of transconjugants by 16S rRNA gene amplicon sequencing

The total bacterial DNA of sorted transconjugants was extracted using a TIANamp Genomic DNA Kit (TianGe, China), following the manufacturer’s instructions. The concentrations of extracted gDNA were measured using a Qubit Flex fluorometer with a dsDNA HS assay kit (Invitrogen, United States). 16S rRNA gene libraries were constructed by amplification of the V3-V4 region of the bacterial 16S rRNA gene using the primer pair 341F (5′-CCTACGGGGNGGCWGCAG-3′) and 805R (5′-GACTACHVGGGGTATCTAATCC-3′). The purified amplicons from each reaction mixture were pooled and sequencing was performed using a MiSeq System (Illumina) according to the manufacturer’s instructions. The raw sequencing data were preprocessed by removing adapter and primer sequences using “cutadapt” (version1.18) [25]. Trimmed reads were denoised and assembled into amplicon sequence variants (ASV) using QIIME2 in conjugation with the R-based DADA2 package with default parameters [26]. Taxonomy was assigned against the SILVA database (v138.1) [27]. The maximum-likelihood phylogenetic tree was built using MEGA (version 11) [28] under the Kimura 2-parameter model. All 16S rRNA gene amplicon sequence data were deposited in the NCBI under the accession number BioProject PRJNA1069862 (Biosample accession No. SAMN41007016 to SAMN41007023).

### Whole-genome sequencing of *Providencia stuartii* transconjugants

Whole-genome sequencing was performed on 11 randomly selected *P. stuartii* transconjugants using the HiSeq System (Illumina). Genomic DNA was extracted using the TIANamp genomic DNA extraction kit (TIANGEN, China) and the concentrations of DNA were quantified using a Qubit Flex fluorometer with a dsDNA HS assay kit (Invitrogen, United States). DNA libraries were prepared using an Annoroad Universal DNA Library Prep Kit V2.0 (AN200101-L) according to the manufacturer’s guidelines, and 150- bp paired-end sequencing was performed on the HiSeq X Ten platform (Illumina). Raw Illumina reads were filtered with Trimmomatic (Galaxy Version 0.38.1) and assembled into contigs using a SPAdes genome assembler (Galaxy Version 3.15.4) with default parameters. The presence of antibiotic resistance genes and plasmid incompatibility types were identified using staramr (Galaxy Version 0.10.0+galaxy1). All sequencing data are available in the NCBI Sequence Read Archive under BioProject accession number PRJNA1069862 (BioSample accession number SAMN40613298 to SAMN40613308).

### Phylogenic analysis of *P. stuartii*

A total of 85 *P. stuartii* were downloaded from the NCBI taxonomy database on 5 December 2023. Species-wide relatedness analysis of 96 global *P. stuartii* isolates (included 11 *P. stuartii* transconjugants isolated from this study) was performed using Parsnp (version 1.5.6) to create a core genome alignment [29]. A maximum likelihood phylogenetic tree was then constructed based on the filtered core-genome SNPs using FastTree (v.2.11.11) [30]. The phylogenetic trees were visualized and annotated using iTOL platform.

### Stability and transferability of newly formed pA/C_MCR-8 transconjugants

For the stability assays: to examine the stability of plasmid pAC_MCR-8 in the new *P. stuartii* hosts, we conducted 15-day serial passages of 11 transconjugants in the absence or presence of 2 mg/l colistin. Briefly, overnight cultures were diluted (1:200) into 200 μl fresh LB with or without colistin (2 mg/l) and re-incubated for 24 hours. The bacterial samples were collected at 0, 3, 6, 9, 12, 15, and 18 days until no green cell was detectable. The presence of *gfp*-tagged plasmid pAC_MCR-8 was measured by flow cytometry as described above. All experiments were conducted in three biological independent replicates.

For the conjugation assays: to investigate the transferability of newly formed *P. stuartii* transconjugants, we selected four clinical meropenem-resistant *K. pneumoniae* strains (Kp85, Kp2069, Kp1106, Kp2058) and five clinical cefotaxime-resistant *Escherichia coli* strains (Ec710, Ec728, Ec736, Ec754, Ec2037) as the recipient strain, and 11 *P. stuartii* transconjugants as donors for conjugation assays. In brief, donor and recipient cells were grown at exponential phase before mixing at a 1:1 ratio in LB and co-incubated overnight at 37°C. After 16 hours, the mixtures were serially diluted and plated on selective UTI agar plates containing colistin (2 mg/l) with meropenem (0.5 mg/l), tigecycline (8 mg/l), or cefotaxime (2 mg/l), when appropriate. The resulting transconjugants were verified by PCR for *mcr-8* and *gfp* genes, and species were identified by 16S rRNA gene amplicon and Sanger sequencing. Finally, we calculated the conjugation frequencies per recipient following the equation below: 


(1) \begin{equation*} transfer\ frequency=\frac{CFU\ (transconjugants)}{CFU\ (recipients)} \end{equation*}

### Fitness evaluation of the pAC_MCR-8 plasmid in different bacterial species

A total of 20 pAC_MCR-8 positive transconjugants were subjected for growth and fitness analysis. This included 11 *P. stuartii*, five *E. coli*, and four *K. pneumoniae* transconjugants (Supplementary TableS1), with their respective plasmid-free recipients. 11 respective plasmid-free *P. stuartii* were obtained from the above stability experiments with plasmid loss. Overnight liquid cultures of transconjugant strains were diluted into LB broth to an OD_600nm_ of 0.1 and incubated in a microplate reader at 37°C for 24 hours. The bacterial OD_600nm_ were measured every hour by SpectraMax iD3 (Molecular Devices, United States). All experiments were performed in triplicate and results represent the average of three experiments. The growth characteristics of bacterial growth were analyzed by the Growthcurver R packages [24] that provides several growth curve metrics including the growth rate (*r*), the carrying capacity (*k*), and the area under the curve (AUC). The project Growthcurver’s home page is available at (http://github.com/sprouffske/growthcurver). We calculated the relative *r*, *k,* and AUC by dividing the average value of each parameter for plasmid-carrying transconjugant to its respective plasmid-free recipient strain, using the following equation:


(2) \begin{equation*} relative\ fitness=\frac{r,k\ or\ AUC\ \left(\mathrm{plasmid}-\mathrm{carrying}\ \mathrm{strain}\right)}{r,k\ or\ AUC\ \left(\mathrm{plasmid}-\mathrm{free}\ \mathrm{strain}\right)} \end{equation*}

### Statistical analysis

The data analysis was performed using GraphPad Prism (8.3.1), and all data shown in plots are represented as mean of three replicates ± SEM. Significant differences were determined using the Kruskal-Wallis test and Mann–Whitney test, as appropriate. The significance level was set to .05 and the exact *P* values are shown in the Figs.

## Results

### High-throughput analysis of the transfer of AMR plasmid in gut microbiome

**Figure 1:**
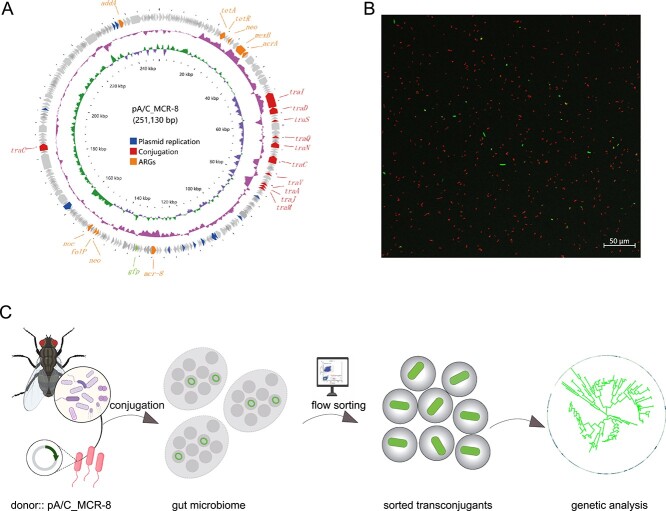
(A) The complete genetic sequence of plasmid pA/C_MCR-8 with the *gfpmut* gene inserted using the CRISPR-Cas gene editing tool. The plasmid map was visualized by CGview (Proksee). Several essential genes were listed on the plasmid such as genes involving in plasmid replication/maintenance, conjugation, and ARGs. The first two inner circles show the GC skew (+ green, −purple) and GC content (pink), respectively. (B) the microscopy image of mating cultures initiated by co-incubating the pA/C_MCR-8-positive donor bacteria and gut microbiota of houseflies for 16 h. The red cells were the donor *E. Coli* MG1655 labelled with a *mCherry* gene and the green cells were transconjugants with newly acquired *gfp*-tagged pA/C_MCR-8 plasmid, whereas the recipients were in the dark background with no fluorescence. (C) the schematic of experimental setup for the transfer of pA/C_MCR-8 within gut microbiota extracted from houseflies.

To investigate the potential contribution of houseflies to the transmission of ARGs, we developed a suite of combined *in vivo* and *in vitro* experimental models to study the transfer of an AMR plasmid within gut microbiota of houseflies. A focus AMR plasmid pA/C_MCR-8 (originated from strain KP22 from chicken cloacae) [31] was selected for this study on the basis of (*i*) the plasmid has conjugative machinery able to horizontally transfer among different bacteria; (*ii*) the plasmid carry resistance gene *mcr-8*, responsible for the resistance to the last-resort antibiotic colistin. For *in vitro* model, gut microbiota collected from five groups of houseflies were challenged with a *gfp*-tagged pA/C_MCR-8-positive donor strain *E. coli* MG1655 [32] (Fig. 1a), and the plasmid transfer rates were determined by flow cytometry (Fig. 1c), as described in our previous study [21]. The ratio of *gfp*-expressing transconjugants in all droplets varied in five independent mixed cultures (Supplementary Fig. S3), ranging from 4.1 × 10^−3^ to 5.0 × 10^−3^ per cell, suggesting that plasmid pA/C_MCR-8 has capable of rapid and efficient transfer within gut microbiome of houseflies. The conjugative events were also observed using confocal laser scanning microscope, specifically, the presence of *gfp*-expressing cells (green; Fig. 1b) demonstrates that plasmid pA/C_MCR-8 has successfully transferred to the new bacterial hosts.

We conducted the colonization experiment using pA/C_MCR-8-positive *E. coli* MG1655 in an *in vivo* housefly model. The *mCherry*-expressing donor strain was detectable in the intestines of houseflies at day 3 (mean = 4.6%) and day 5 (mean = 0.2%), but became completely undetectable after 5 days (Supplementary Fig. S4), suggesting that colonization resistance and limited growth within the anaerobic gut environment may hinder the transfer of plasmid pA/C_MCR-8 across gut microbiome of houseflies. Indeed, no transfer events were detected by flow cytometry, likely due to the *in vivo* transfer rate being below the detection limit (less than 0.001% of *gfp*-positive transconjugants in total cells).

### Phylogenetic analysis of transconjugal pools

**Figure 2:**
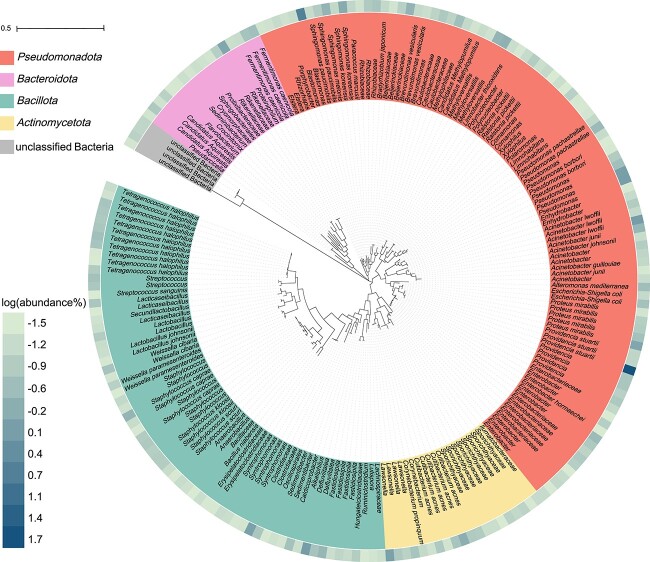
The phylogenetic tree shows 200 identified ASVs in transconjugant pools sorted from the gut microbiota of houseflies (with the mean abundance >0.03%). The colors of the branches mark different phylogenetic groups. The outermost circle at the periphery of the tree is a heat map showing the log_10_-transformed relative ASV abundance in the transconjugal pools. ASV, amplicon sequence variants.

To further identify the new bacterial hosts of plasmid pA/C_MCR-8, *gfp*-expressing transconjugants from the above *in vitro* experiments were sorted and subjected to16S rRNA gene amplicon sequencing. All green cells were observed on the selective agar plates (Supplementary Fig. S3), indicating the high purity of sorted transconjugal cells. The phylogenetic analysis of the transconjugant cells indicates that plasmid pA/C_MCR-8 could be transferred broadly across gut microbiota of houseflies (Fig. 2). The primary bacterial hosts of plasmid pA/C_MCR-8 belong to the phyla *Pseudomonadota* and *Bacillota*, which comprises 75.9% and 11.8% of 200 transconjugal ASVs with ASV abundance threshold >0.03%, respectively. The remaining two phyla consist mainly of *Actinomycetota* and *Bacteroidota*. The major bacterial genus of the transconjugant pools was *Providencia* with an abundance value of 24.8–70.0% (mean = 51.1%), which illustrates that *Providencia* is the main host for plasmid dissemination in the gut microbiota of houseflies, followed by *Blastomonas* genus with abundance 0.05%–13.2% (mean = 5.2%). This possibly due to relative high abundance (approx.12.9%) of *Providencia* detected in the gut microbiome of houseflies (belonging to the top three bacterial genera, Fig.S5), which is in line with previous findings [33]. Because *Providencia* is an opportunistic pathogen known to cause a range of human infections including urinary tract infections and bacteremia [34, 35], the transfer of *mcr-8*-postive plasmid among *Providencia* species from houseflies can have notable implications for human health.

### Phylogenetic association of *Providencia* transconjugant with human health

**Figure 3:**
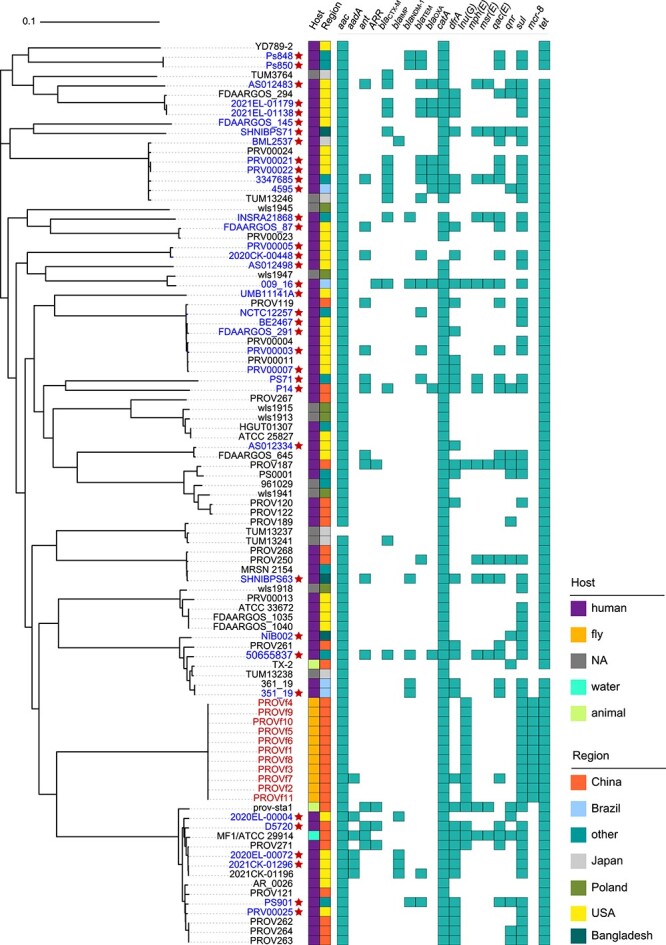
The maximum-likelihood phylogenic tree of 95 global *Providencia stuartii*, based on the core genomic SNPs of *P. Stuartii* strains. The *P. Stuartii* strains obtained from this study were shown in red strain IDs. The presence of ARGs, host information, and countries were marked by blocks. *Providencia* strains associated with infection diseases were noted with red stars and strain IDs with light blue color. The GenBank accession number and genotypic information for these 95 *P. stuartii* are also available in dataset 1.

To further characterize the genetic association of the *Providencia* transconjugants of houseflies with those from other settings, a total of 11 *Providencia* transconjugants were isolated from different single colonies and subjected to whole-genome sequencing. Based on genomic single nucleotide polymorphism (SNP) analysis, all *mcr-8*–positive *Providencia* transconjugants showed 98.9%–99.0% average nucleotide identity identity to the reference strain *P. stuartii* ATCC29914 (Genbank number GCF_010669105, Supplementary Table S2), suggesting that these *Providencia* transconjugants belong to the bacterial species *P. stuartii*. Furthermore, to better understand the genetic closeness of *Providencia* transconjugants from houseflies with worldwide spread of *P. stuartii* genomes, a total of 84 *P. stuartii* genomes downloaded from the NCBI RefSeq collection (Supplementary Dataset 1). Based on bacterial core-genome SNPs, the maximum-likelihood phylogenetic tree was constructed, revealing a close genetic affinity between 11 *P. stuartii* transconjugants obtained from houseflies and those originating from humans (Fig. 3). This genetic similarity highlights the potential for cross-species transmission between *Providencia* isolates from houseflies and those from humans. Furthermore, the genetic characteristics of ARGs and plasmids were analyzed, and all isolates were found to be multidrug resistant and harbored more than three different classes of ARGs, with particularly high incidences of the resistance genes *aac* (94/95), *cat* (92/95), and *tet* (93/95) (Fig. 3). The presence of *bla*_IMP_, *bla*_NDM_, *bla*_OXA_, and *mcr-8* is of serious concern, as these genes confer resistance to clinical last-resort antibiotics (e.g. carbapenems and colistin). Taken together, these findings strongly align with those of previous studies indicating that houseflies play a potentially significant role as vehicles for the transmission of ARGs and human-associated bacterial pathogens [36, 37] and pose a potential risk for further dissemination to humans via food chains.

### Transferability and fitness of new *P. Stuartii* transconjugants

**Figure 4:**
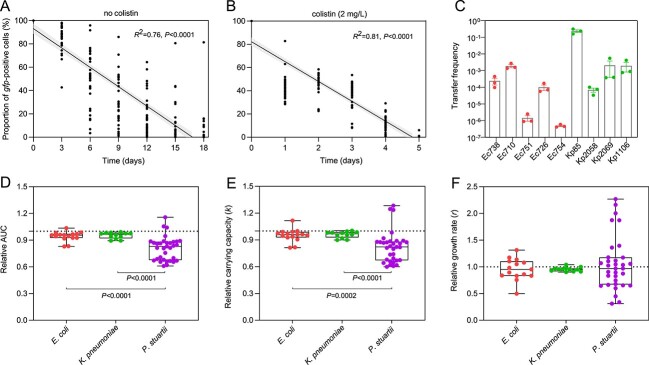
Stability and transferability of pA/C_MCR-8 in *P. Stuartii* transconjugants. (A and B) the persistence of pA/C_MCR-8 in 11 isolated *P. stuartii* transconjugants under the presence or absence of colistin, respectively. Simple linear regressions with 95% confidence bands were added, indicating the statistical significance of plasmid loss over time. (C) the transfer of plasmid pA/C_MCR-8 from donor strain *P. stuartii* to 9 clinically relevant strains (five *E. coli* and four *K. pneumoniae*). The transfer frequencies are calculated by the number of transconjugants per recipient. The colors of dots represent different bacterial species. (D–F) Relative values of growth curve parameters: area under the growth curve (AUC), carrying capacity (*k*), and maximum growth rate (*r*_max_), represented as boxplots for different bacterial hosts. Horizontal lines inside boxes indicate median values, the upper and lower hinges correspond to the 25th and 75th percentiles, and whiskers extend to observations within minimum and maximum range. Dots represent each relative value, when the relative values <1 indicate a reduction in these parameters associated with plasmid acquisition. Three biological replicates of the growth curves were performed for the wild-type isolates and their respective transconjugants.

We further investigated the stability and transferability of the pA/C_MCR-8 plasmid in *P. stuartii* transconjugants. A total of 11 *P. stuartii* transconjugants (PROVf1 to PROVf11) were sequentially passaged in LB broth with or without colistin (2 mg/l) for 18 days. The declining simple linear regressions indicate the rapid loss of the pA/C_MCR-8 plasmid in *P. stuartii*, regardless of the presence of antibiotics (Fig. 4A and B). In the absence of colistin, the percentages of pA/C_MCR-8–positive bacterial cells were gradually decreased and dropped to 1.0% at day 18 (Fig. 4A and Supplementary Fig. S6). In contrast, the percentage of plasmid-positive cells was deceased to 1.0% in 5 days, indicating the higher plasmid loss rate in the presence of antibiotics compared to the nonantibiotic group (Fig. 4B and Supplementary Fig. S7). This observation may be attributable to the intrinsic resistance of *P. stuartii* to colistin (minimum inhibitory concentration [MIC] > 128 mg/l, Supplementary Table S1) [35]. Furthermore, *P. stuartii* transconjugants readily transfer pA/C_MCR-8 to other clinically relevant pathogens, including four *Klebsiella pneumoniae* (Kp85, Kp1106, Kp2058, Kp2069) and five *E. coli* strains (Ec710, Ec728, Ec736, Ec754, Ec2037). The transfer rates between different donor–recipient pairs were significantly varied (Kruskal–Wallis test, *P* = .002), ranging from 2.3 × 10^−1^ to 5.9 × 10^−7^ per recipient (Fig. 4C).

To analyze the distribution of the fitness effect of plasmid pA/C_MCR-8 in different bacterial hosts, we used several growth parameters as proxies of bacterial fitness. Indeed, fitness effects of plasmid pA/C_MCR-8 varied greatly among the isolates in the collection, ranging from benefits to burdens (Fig. 4D–F). For instance, the acquisition of plasmid pA/C_MCR-8 appears to confer a higher fitness burden in the *P. stuartii* hosts, as indicated as lower *k* and AUC values compared to the plasmid in *K. pneumoniae* and *E. coli* hosts (Mann–Whitney test, *P* < .05). The observed fitness depicted in *P. stuartii* strains may contribute to an explanation of the instability of pA/C_MCR-8 plasmid (Fig. 4A and B). Collectively, these results indicate that the presence of houseflies as carriers of both ARGs and clinically relevant pathogens further emphasizes their role as potentially critical vectors for the spread of AMR.

## Discussion

AMR is a global problem that is primarily exacerbated by the overuse and misuse of antibiotics in human medicines and agriculture [38]. However, there are additional factors that contribute to the spread of AMR, including substandard sanitation and environmental contamination, particularly in low- and middle- income countries (LMICs) [39, 40]. Numerous studies have showed that flies can serve as mechanical vectors, which may facilitate the transmission of ARGs between the environment and humans [41–43]. This finding underscores the importance of understanding and mitigating the role of insects in the spread of AMR, especially in environments where there is close interaction between humans, animals, and insects. However, these studies have focused on identifying ARGs in flies, but the transfer dynamics of AMR plasmids within their gut microbiota have not been investigated. Moreover, these previous studies lack rigorous evidence obtained through whole-genome sequencing to verify the association of pathogenic bacteria with interactions between flies and human hosts. In this study, we investigated the *in situ* transfer of the *mcr-8_*positive plasmid pA/C_MCR-8 in gut microbiota of houseflies with state-of-art methodologies and demonstrate that the plasmid pA/C_MCR-8 exhibits broad transmission potential within gut microbiota, with transfer rates ranging from 4.1 × 10^−3^ and 5.0 × 10^−3^ per cell. The plasmid pA/C_MCR-8 is capable of transferring across various bacterial phyla, *Pseudomonadota* and *Bacillota* in particular. The further analysis of the phylogenetic relationships of plasmid key hosts, particularly *P. stuartii*, revealed a close genetic identity to *P. stuartii* pathogens known to cause human infections (Figs. 2 and 3). This genetic similarity suggests a potential link between environmental reservoirs, such as houseflies carrying pA/C_MCR-8, and clinical isolates of *P. stuartii* responsible for human infections. *Providencia* bacteria originating from flies also play a key role in transferring the *mcr-8* gene to clinically relevant pathogens (i.e. *K. pneumoniae*, Fig. 4C), Our findings clearly support the transfer potential of clinically relevant pathogens and ARGs from houseflies to humans, highlighting the importance of sanitation and hygiene in the prevention of AMR from environmental sources, such as houseflies [44].

Although the relationship between flies and AMR is unsurprising [45], we report the conjugative transmission of an AMR plasmid within the gut microbiota of flies and potentially allowing for the transfer of ARGs to clinically relevant pathogenic bacteria through conjugation. After the introduction of a plasmid-mediated *mcr-8* gene into gut microbiota, it can rapidly propagate throughout the microbiome of flies, highlighting the potential for widespread transmission of AMR. Moreover, the transconjugant *P. stuartii* belonging to the Gram-negative *Enterobacteriaceae* family are closely related to disease-associated *P. stuartii* (Fig. 3), which is a common cause of hospital-acquired infections, including urinary tract infections [46], neonatal sepsis [47], and conjunctivitis [48] and meningitis after neurosurgery [49]. These organisms typically exhibit multidrug resistance to most commonly used antimicrobials including gentamicin [50], tetracycline [51], and meropenem [52]. We also report that transconjugant *P. stuartii* isolated from fly gut microbiota were able to transfer plasmid pA/C_MCR-8 to pathogenic *K. pneumoniae*, indicating the significant role of houseflies as a potential source of multidrug-resistant (MDR) pathogens in both hospital settings and communities. Furthermore, the persistence of pA/C_MCR-8–positive *E. coli* strain in the housefly intestine for 5 days (Supplementary Fig. S3) is consistent with previous findings [53] and indicates that the intervals are sufficient for transmission to human environments. The intervention of fly control measures (e.g. onsite sanitation) may reduce the infection risks posed by flies [54], suggesting a need to design sanitation strategies to prevent the transmission of enteric pathogens.

Our study, along with other research, clearly emphasizes that the challenges of AMR go beyond clinical settings and are aligned with the behavior of flies [7, 55, 56], thus creating further risks to human and environmental health. The behavior of flies, including their feeding habits and movement patterns, can contribute to the transmission of pathogenic bacteria and ARGs [57]. For instance, flies feed on waste materials such as feces, garbage, and decaying matter, where they can pick up MDR bacteria and further transmit them to surface, food, and humans [58, 59]. However, in many communities of residents with low socioeconomic status, particularly in LMICs [60, 61], insect populations are often poorly controlled in healthcare settings and inadequate waste management leads to reservoirs of MDR bacteria that enable flies to rapidly distribute MDR isolates across One Health settings [43, 62]. A population modelling study has estimated that fly populations may increase by 156.0%–244.0% by 2080, due to the changes caused by the warming climate [63]. The proliferation of fly populations and its impact on AMR is concerning because it could exacerbate the current global AMR threat scenario, particularly in LMICs.

Our study unveiled the transfer dynamics and breadth of an AMR plasmid across gut microbial communities of houseflies, with potential implications for global public health. However, it is important to acknowledge some limitations in our study. First, we limited ourselves to examining the *in vitro* transfer of the AMR plasmid, as no transconjugant was observed in the *in vivo* model. This discrepancy might be attributed to host-specific factors or environmental conditions within the gut, such as variations in pH across the alimentary canal of houseflies [64] and in temperature and the anaerobic environment, which could hinder both the colonization of the donor strain and successful plasmid transfer. Second, our observations primarily explored the transfer dynamics of a specific AMR plasmid, pA/C_MCR-8, and future research is needed to comprehensively assess the transferability of other AMR plasmids across diverse microbial communities. Additionally, further exploration of the potential impact of environmental factors such as diet and antimicrobial stress is necessary to understand the potential impact of these factors on plasmid transfer efficiency. This broader investigation would provide more comprehensive understanding of the environmental drivers influencing the dissemination of AMR across One Health settings.

In conclusion, our findings indicate that houseflies serve as biological vectors for the transmission of clinically important ARGs through gut microbiota. Through our investigation of the host characteristics of the pA/C_MCR-8 plasmid, including its broad host ranges, association with human pathogens, and persistence in the gut microbes of houseflies, we have highlighted the potential risks associated with those common insects. The findings of the curret study emphasize the urgent need for comprehensive strategies to mitigate the spread of AMR, including enhanced surveillance, improved sanitation practices, and control of housefly populations. By addressing these issues, we can contribute to the efforts aimed at reducing the global threat of AMR and protecting public health.

## Supplementary Material

Final_supplementary_materials_wrae128

dataset_1_wrae128

## Data Availability

All data generated or analyzed during this study are included in the main text and Supplementary files of this article. The 16S rRNA gene amplicon sequencing data generated in this study have been deposited in the NCBI Sequence Read Archive under the accession numbers PRJNA1069862 (Biosample accession numbers SAMN41007016 to SAMN41007023). The whole-genome sequencing data of 11 *P. stuartii* generated in this study have been deposited in the NCBI database with accession number PRJNA1069862 (BioSample accession numbers SAMN40613298 to SAMN40613308).
