# Supplementary material for: Housefly gut microbiomes as a reservoir and facilitator for the spread of antibiotic resistance
Source: ISME J. 2024 Jul 20;18(1):wrae128. doi: 10.1093/ismejo/wrae128 (PMC11456846; doi:10.1093/ismejo/wrae128)
Supplement: Final_supplementary_materials_wrae128 [file final_supplementary_materials_wrae128.docx]

Supplementary Materials for

**Housefly gut microbiomes serve as a reservoir and facilitator for the spread of antibiotic resistance**

Gande Hao^1,2^, Zhenyan Lin^1^, Lingshuang Zeng^1^, Hui Deng^2^, Timothy R. Walsh^3^, Shungui Zhou^1*^, Qiu E. Yang^1*^

Corresponding author: prof. Qiu E Yang ([qiueyang2014@163.com](mailto:qiueyang2014@163.com)) and prof. Shungui Zhou ([sgzhou@fafu.edu.cn](mailto:sgzhou@fafu.edu.cn))

**The PDF file includes:**

Tables S1 to S2

Figs. S1 to S7

References

**Supplementary Table S1-S2**

**Table S1** The list of strains used for conjugation and stability assays

| **Strain ID** | **Species** | **Antibiotic resistance*** | **Origin** | **Strain info.** | **MIC of Coli (mg/L, after plasmid loss)** |
| --- | --- | --- | --- | --- | --- |
| PROVf1 | *P. stuartii* | Coli^R^ | houseflies | Transconjugant | >128 |
| PROVf2 | *P. stuartii* | Coli^R^ | houseflies | Transconjugant | >128 |
| PROVf3 | *P. stuartii* | Coli^R^ | houseflies | Transconjugant | >128 |
| PROVf4 | *P. stuartii* | Coli^R^ | houseflies | Transconjugant | >128 |
| PROVf5 | *P. stuartii* | Coli^R^ | houseflies | Transconjugant | >128 |
| PROVf6 | *P. stuartii* | Coli^R^ | houseflies | Transconjugant | >128 |
| PROVf7 | *P. stuartii* | Coli^R^ | houseflies | Transconjugant | >128 |
| PROVf8 | *P. stuartii* | Coli^R^ | houseflies | Transconjugant | >128 |
| PROVf9 | *P. stuartii* | Coli^R^ | houseflies | Transconjugant | >128 |
| PROVf10 | *P. stuartii* | Coli^R^ | houseflies | Transconjugant | >128 |
| PROVf11 | *P. stuartii* | Coli^R^ | houseflies | Transconjugant | >128 |
| EC710 | *E. coli* | CTX^R^ | Human | recipient | NA |
| EC728 | *E. coli* | CTX^R^ | Human | recipient | NA |
| EC736 | *E. coli* | CTX^R^ | Human | recipient | NA |
| EC754 | *E. coli* | CTX^R^ | Human | recipient | NA |
| EC2037 | *E. coli* | CTX^R^ | Human | recipient | NA |
| Kp1106 | *K. pneumoniae* | Mero^R^ | Human | recipient | NA |
| Kp2058 | *K. pneumoniae* | Mero^R^ | Human | recipient | NA |
| Kp2069 | *K. pneumoniae* | Mero^R^ | Human | recipient | NA |
| Kp85 | *K. pneumoniae* | Tig^R^ | Human | recipient[1] | NA |

*Coli^R^: colistin; CTX^R^: cefotaxime; Mero^R^: meropenem; Tig^R^: tigecycline. NA, non-available.

**Table S2** ANI scores of 11 *Providencia* transconjugants to a reference strain and their associated resistance genes

| **ID** | **Species** | **Reference strain^*^** | **ANI (%)** | **Resistance genotypes** |
| --- | --- | --- | --- | --- |
| PROVf1 | *P. stuartii* | ATCC29914 | 98.96 | *aac(2')-Ia, aph(3'')-Ib, aph(6)-Id, catA3, floR, lnu(G), mcr-8, sul2, tet(A), tet(B)* |
| PROVf2 | *P. stuartii* | ATCC29914 | 98.98 | *aac(2')-Ia, aph(3'')-Ib, aph(6)-Id, catA3, floR, lnu(G), mcr-8, sul2, tet(A), tet(B)* |
| PROVf3 | *P. stuartii* | ATCC29914 | 99.01 | *aac(2')-Ia, aph(3'')-Ib, aph(6)-Id, catA3, floR, lnu(G), mcr-8, sul2, tet(A), tet(B)* |
| PROVf4 | *P. stuartii* | ATCC29914 | 98.94 | *aac(2')-Ia, aph(3'')-Ib, aph(6)-Id, catA3, floR, lnu(G), mcr-8, sul2, tet(A), tet(B)* |
| PROVf5 | *P. stuartii* | ATCC29914 | 99.00 | *aac(2')-Ia, aph(3'')-Ib, aph(6)-Id, catA3, floR, lnu(G), mcr-8, sul2, tet(A), tet(B)* |
| PROVf6 | *P. stuartii* | ATCC29914 | 98.97 | *aac(2')-Ia, aph(3'')-Ib, aph(6)-Id, catA3, floR, lnu(G), mcr-8, sul2, tet(A), tet(B)* |
| PROVf7 | *P. stuartii* | ATCC29914 | 99.00 | *aac(2')-Ia, aph(3'')-Ib, aph(6)-Id, catA3, floR, lnu(G), mcr-8, sul2, tet(A), tet(B)* |
| PROVf8 | *P. stuartii* | ATCC29914 | 99.02 | *aac(2')-Ia, aph(3'')-Ib, aph(6)-Id, catA3, floR, lnu(G), mcr-8, sul2, tet(A), tet(B)* |
| PROVf9 | *P. stuartii* | ATCC29914 | 98.98 | *aac(2')-Ia, aph(3'')-Ib, aph(6)-Id, catA3, floR, lnu(G), mcr-8, sul2, tet(A), tet(B)* |
| PROVf10 | *P. stuartii* | ATCC29914 | 99.01 | *aac(2')-Ia, aph(3'')-Ib, aph(6)-Id, catA3, floR, lnu(G), mcr-8, sul2, tet(A), tet(B)* |
| PROVf11 | *P. stuartii* | ATCC29914 | 99.01 | *aac(2')-Ia, aac(3)-IV, aadA2, aph(3'')-Ib, aph(3')-Ia, aph(4)-Ia, aph(6)-Id, catA3, dfrA12, floR, lnu(F), lnu(G), mcr-8, qacE, sul1, sul2, tet(A), tet(B)* |

**^*^** The Genbank accession number of ATCC29914: GCF_010669105.1. Two decimals were applied in ANI (%).

**Supplementary Figure S1-S7**

**
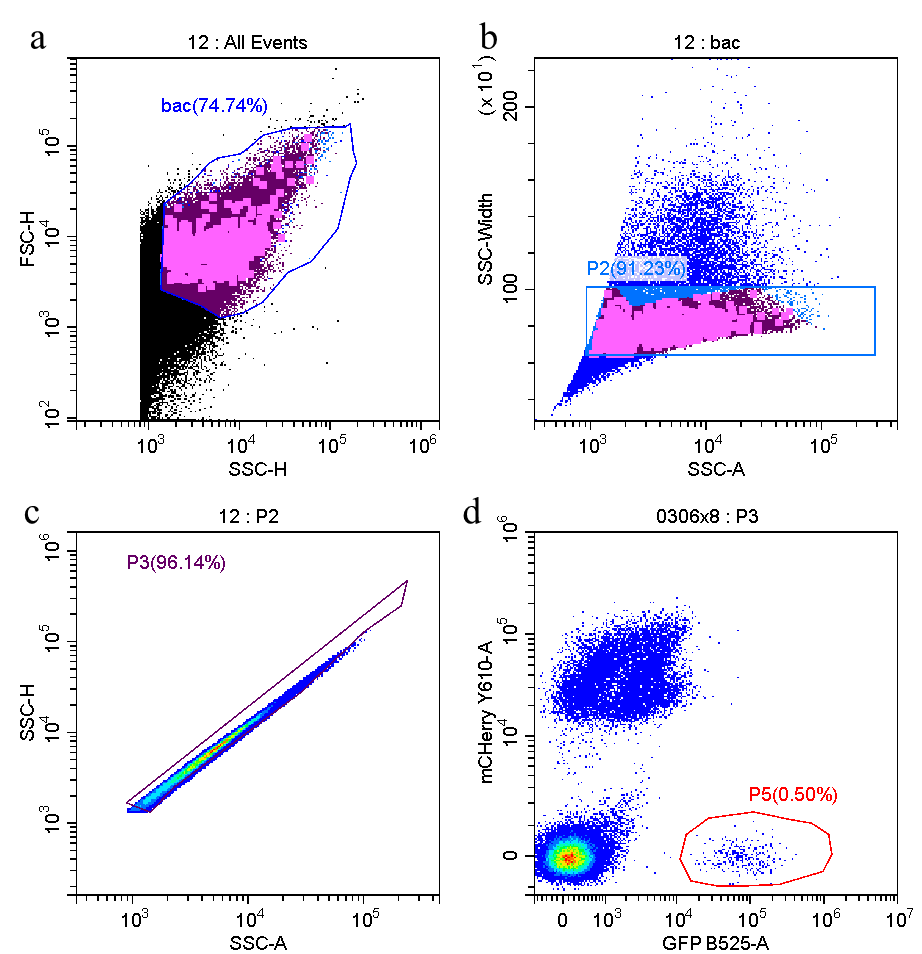
**

**Fig. S1** The gating strategy of sorting the *gfp*-expressing cells from mating mixtures by a cell sorter (cytoFLEX SRT, Beckman, USA). The procedure consists of four consecutive gates with bivariate plots: (a) Gate I was drawn to select only particles of bacterial sizes based on forward and side scatter (FSC-H vs SSC-H); (b-c) Gates II and III were drawn to remove any doublets and select singular bacterial cells using (SSC-W vs SSC-A) and (SSC-H vs SSC-A), respectively; (d) Gate IV was analyzed using B525-A (*gfp*) vs Y610-A (*mCherry*) fluorescence intensity, to distinguish between the donor (*mCherry*, top left), recipient (no fluorescence, bottom left), and transconjugant cells (*gfp*, bottom right). The percentage of *gfp*-expressing cells were circled in pink.

**
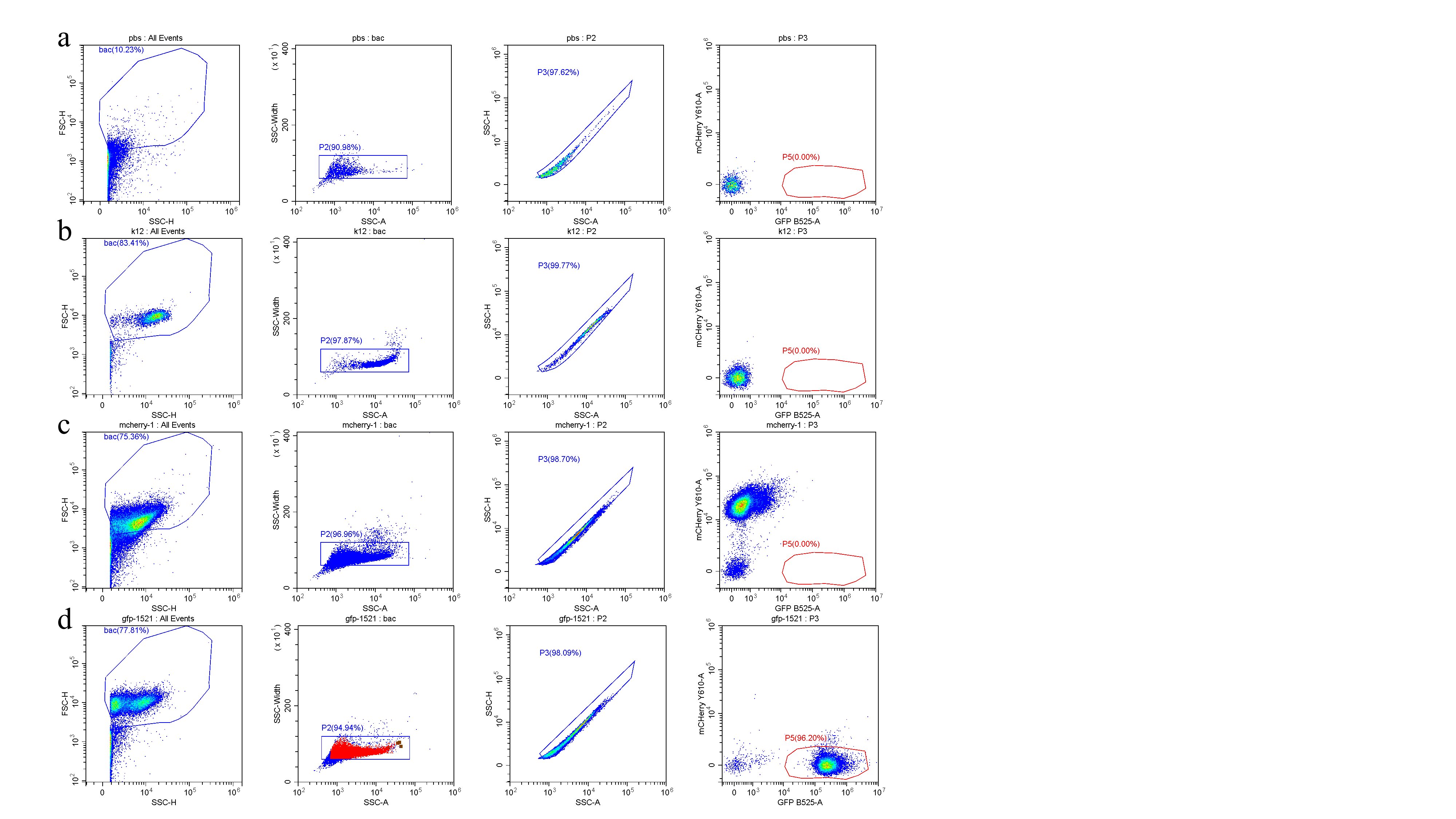
**

**Fig. S2 Establishment of controls for fluorescence detection using flow cytometry (CytoFLEX SRT, Beckman, USA).** (a) non-bacterial control: PBS buffer was used to assess background noise and establish baseline fluorescence levels; (b) non-fluorescent bacterial control: *E. coli* MG1655 strain without fluorescence gene served as a negative control, to determine the baseline fluorescence level; (c) *mCherry*-positive control: *E. coli* MG1655 donor bacteria with mCherry fluorescent protein was used to validate the detection capability and intensity of the *mCherry* signal; (d) *gfp*-positive control: Kp85 strain harboring the *gfp*-tagged plasmid pA/C_MCR-8 was used to optimize sorting gate parameters based on GFP signal intensity for effective sorting *gfp*-expressing cells.

**
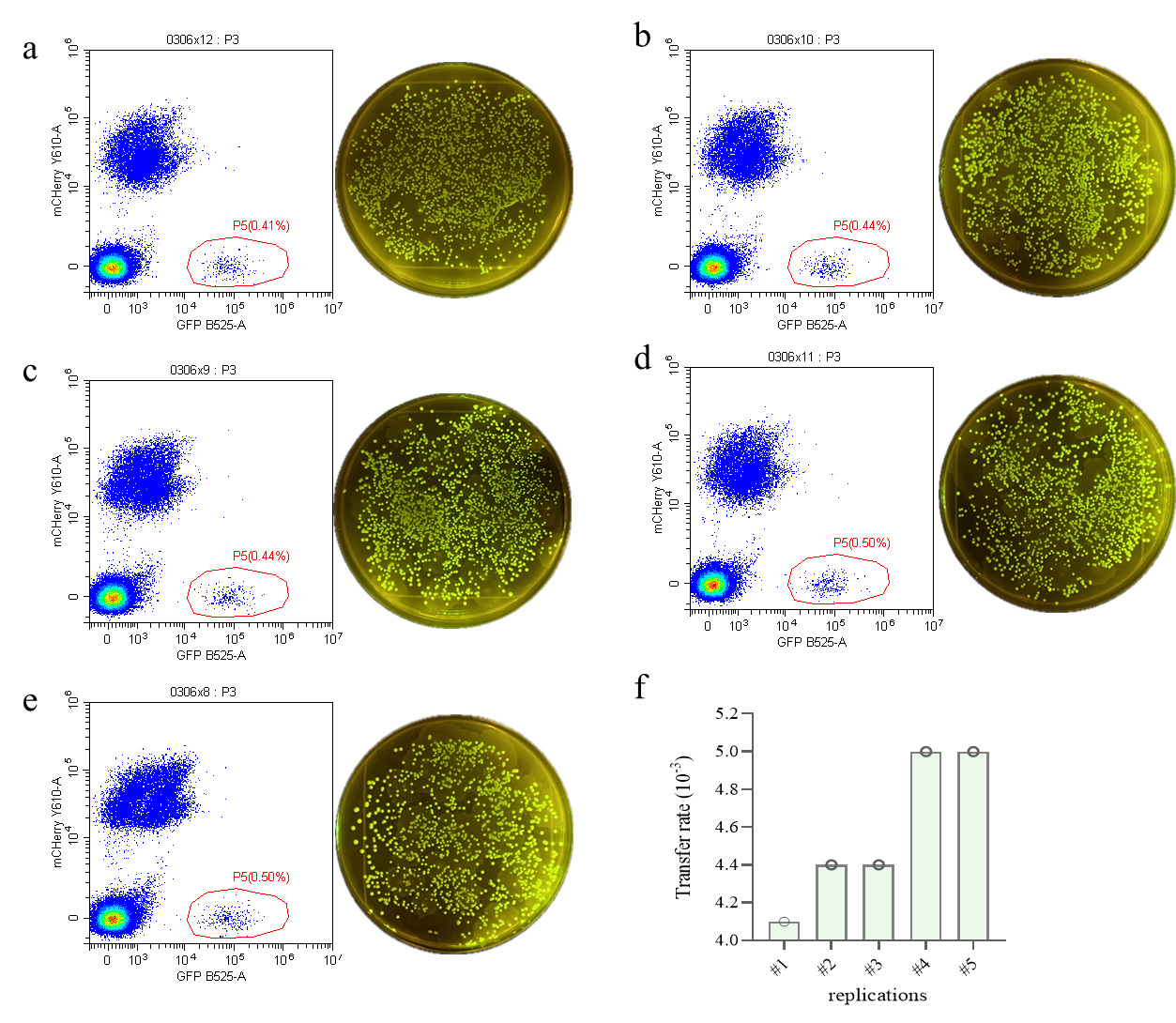
**

**Fig. S3** The flow plots of five independent mating mixtures initiated with gut microbiota of houseflies with the donor strain carrying a *gfp*-tagged pA/C_MCR8 plasmid (*in vitro* model). The gating strategy was indicated in Fig.S1. (a-e) In each flow plot (n=5 replications), about 50 000 events were recorded and the percentage of *gfp*-expressing transconjugants were highlighted with red circle. The green colonies from sorted culture were observed on the agar plates supplemented with 2 mg/L colistin (right), indicating the high purity of the sorted cells. (f) indicating the percentage of *gfp*-positive transconjugants (indicated by the scientific notation) in five replications.

**

**

**Fig. S4** The presence of *E. coli* MG1655 donor (with *mCherry* fluorescence) in the gut microbiota of houseflies (in *in vivo* model). (a) the flow plots indicate the percentage of *mCherry*-positive donor (top left gate) in the gut microbiota of houseflies over five days using the Attune NxT flow cytometer (ThermoFisher, USA). (b) the average percentage of *mCherry*-positive donor bacteria in the gut of houseflies over five days (n=3).





**Fig. S5** A phylogenetic tree displaying the identification of all ASVs (n=217, abundance>0.03%) at phylum level in gut microbiota of *Musca domestica*. Phylogenetic groups are represented by the colors of the branches on the tree. A heatmap circle represents the relative abundance of ASVs in the recipient community. All abundance values have been converted to logarithm base 10 values. ASV: Amplicon Sequence Variants

**
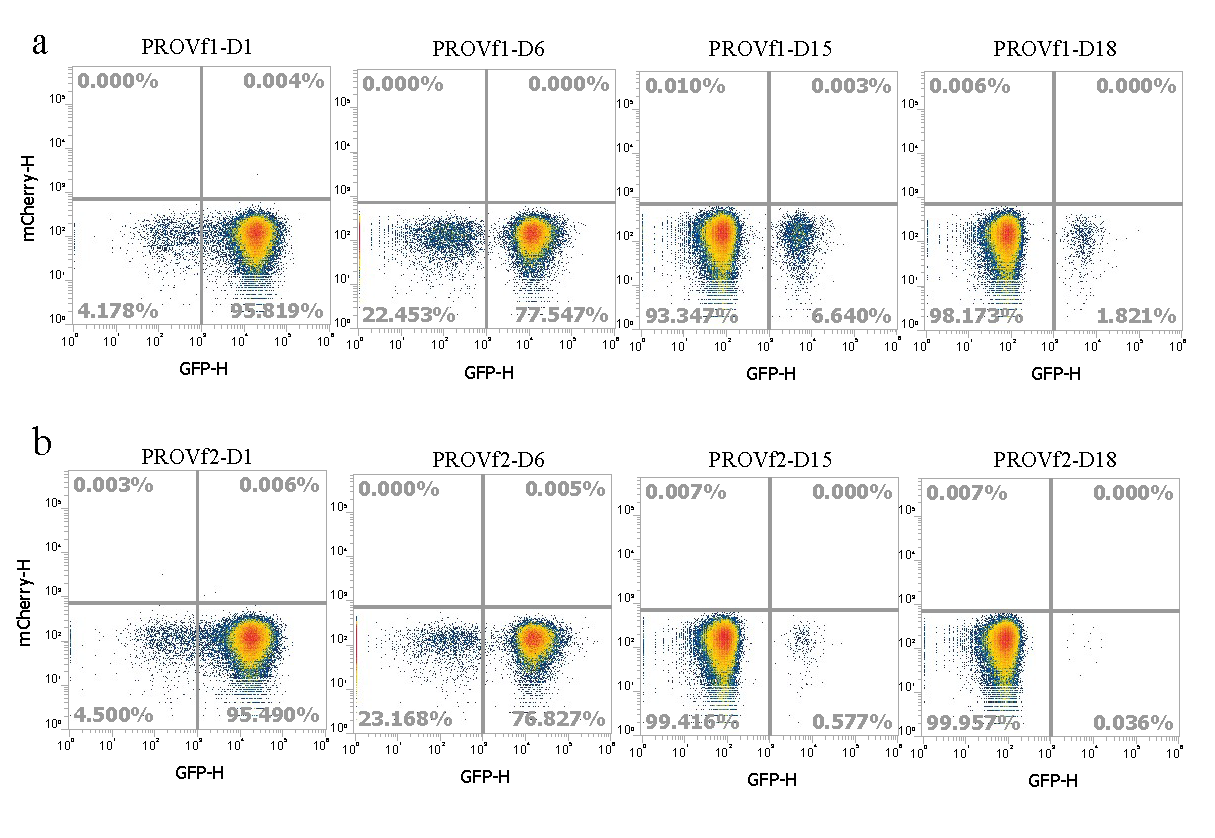
**

**Fig. S6** The representative flow plots to determine the stability of plasmid pA/C_MCR-8 in two *P. stuartii* strains by flow cytometry (Attune NxT, ThermoFishe, USA). (a-b) showing the percentage of *gfp*-expressing cells on 1, 6, 15, and 18 days for strain PROVf1 and PROVf2 transconjugants in the absence of antibiotics, respectively. The percentage of *gfp-*expressing cells was declined over time, indicating the loss and instability of plasmid pA/C_MCR-8 in *P. stuartii* strains.


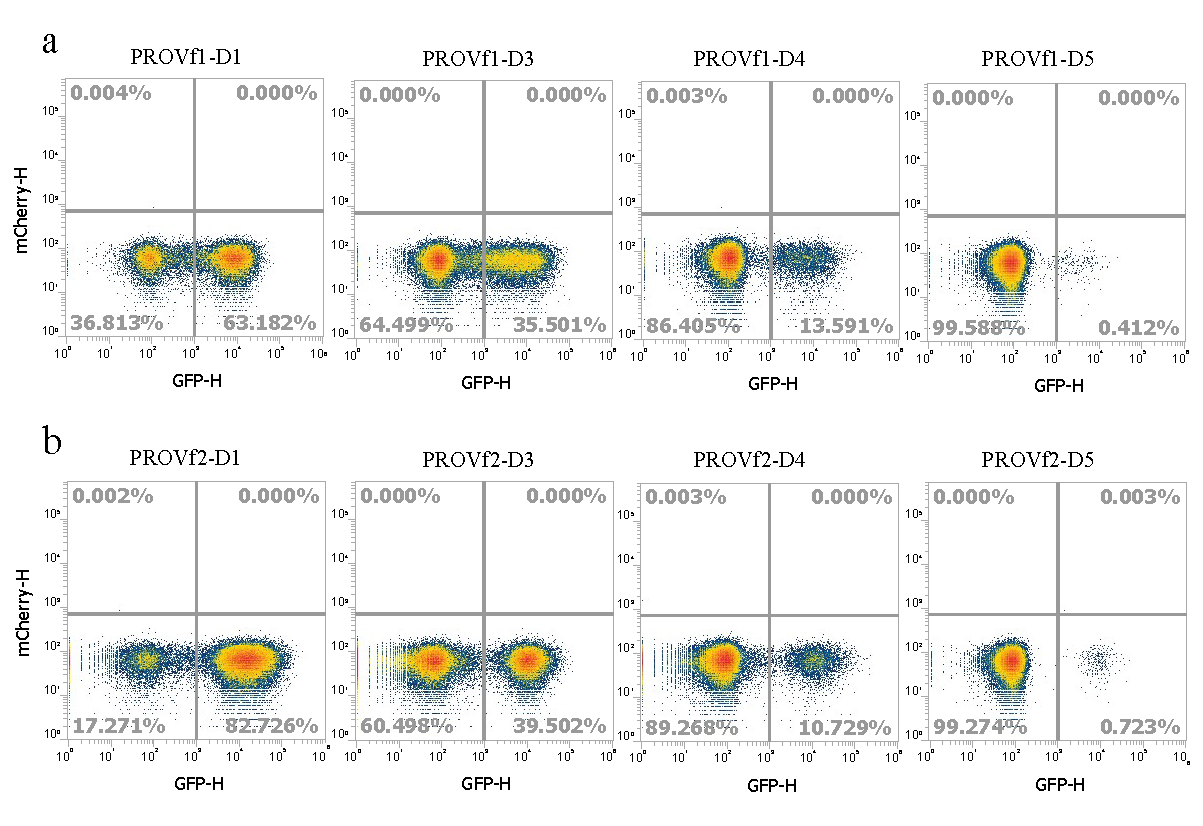


**Fig. S7** The representative flow plots to determine the stability of plasmid pA/C_MCR-8 in two *P. stuartii* strains by flow cytometry (Attune NxT, ThermoFisher, USA). (a-b) showing the percentage of *gfp*-expressing cells on 1, 3, 4, and 5 days for strain PROVf1 and PROVf2 transconjugants in the presence of colistin (2 mg/L), respectively. The percentage of *gfp-*expressing cells was significantly decreased within five days, indicating plasmid pA/C_MCR-8 was instable in *P. stuartii* strains in the presence of colistin.

**Reference**

1. Zhai W, Tian Y, Lu M, Zhang M, Song H, Fu Y, *at al*. Presence of mobile tigecycline resistance gene tet(x4) in clinical klebsiella pneumoniae. *Microbiol Spectrum*. 2021;**10**:e01081-21
